# Inhibition of Platelets by Clopidogrel Suppressed Ang II‐Induced Vascular Inflammation, Oxidative Stress, and Remodeling

**DOI:** 10.1161/JAHA.118.009600

**Published:** 2018-10-24

**Authors:** Xiangbo An, Guinan Jiang, Cheng Cheng, Zhengshuai Lv, Yang Liu, Feng Wang

**Affiliations:** ^1^ Department of Interventional Therapy the First Affiliated Hospital of Dalian Medical University Dalian China; ^2^ Department of Anesthesia the First Affiliated Hospital of Dalian Medical University Dalian China; ^3^ Institute of Cardiovascular Diseases the First Affiliated Hospital of Dalian Medical University Dalian China; ^4^ Center for Clinical Research on Neurological Diseases the First Affiliated Hospital of Dalian Medical University Dalian China

**Keywords:** inflammation, P2Y12, platelet, platelet receptor blocker, vascular inflammation, Vascular Disease, Hypertension, Fibrosis, Inflammation, Platelets

## Abstract

**Background:**

Platelets play a role in promoting inflammatory responses under several disease conditions. Platelets are activated in hypertensive patients. However, the mechanisms responsible for platelet‐mediating vascular inflammation are unknown. The present study investigated the role of platelets in promoting vascular inflammation following angiotensin II (Ang II) stimulation, and the efficacy of antiplatelet intervention.

**Methods and Results:**

Within a mouse model of Ang II infusion (490 ng/kg per min), we measured the portion of P‐selectin–positive platelets and platelet‐monocyte (P‐M) binding in blood samples, and platelet accumulation and P‐M binding in vessels under Ang II stimulation at days 1, 3, and 7. We tested the efficacy of clopidogrel (15 mg/kg per day, followed by 5 mg/kg per day) on Ang II‐induced platelet activation, P‐M binding, vascular platelet accumulation, as well as vascular inflammation and remodeling at day 7 or 14. Clopidogrel reduced platelet vascular deposition (28.7±2.4% versus 18.3±2.9%), suppressed inflammatory cell infiltration (3.6±0.8×10^4^/vessel versus 2.3±1.2×10^4^/vessel) and oxidative stress, and attenuated vascular remodeling and dysfunction (55.0±5.5% versus 84.0±6.0%) following Ang II stimulation at day 7 or 14. Clopidogrel suppressed Ang II‐induced P‐M binding both at circulating (13.4±3.3% versus 5.9±2.7%) and regional (33.4±4.3% versus 11.9±2.7%) levels.

**Conclusions:**

Platelets play a critical role in vascular inflammation under Ang II stimulation, with a marked promotion of P‐M binding as an important mechanism. Clopidogrel prevented vascular inflammation in Ang II‐infused mice.

**Clinical Perspective**

**What Is New?**

- Platelets were accumulated in the vessels following angiotensin II infusion.
- Angiotensin II‐induced elevation in platelet‐monocyte/macrophage binding was observed both in blood and vessels.
- Clopidogrel reduced platelet vascular deposition, suppressed inflammation and oxidative stress, and attenuated vascular remodeling and dysfunction following angiotensin II stimulation.
- Clopidogrel suppressed angiotensin II ‐induced platelet‐monocyte/macrophage binding at both circulating and regional levels.

**What Are the Clinical Implications?**

- In hypertensive patients, platelet‐monocyte/macrophage binding could serve as a useful cellular marker both for the degree of vascular inflammation and systemic inflammation.
- Inhibiting platelet activation during hypertension might be useful as an adjuvant treatment to improve outcomes of antihypertensive treatment.

Hypertension is the major risk factor for cardiovascular disease, kidney disease, and stroke. The renin‐angiotensin‐aldosterone system, particularly angiotensin II (Ang II), contributes to the development of hypertension and is able to induce vascular inflammatory responses, eventually leading to vascular remodeling and dysfunction.^1^, ^2^

Platelets are small, anucleate cells and are produced by bone marrow megakaryocytes, which have important vascular actions involving promotion of atherosclerotic growth, plaque instability, and thrombosis. The pro‐inflammatory actions of platelets on heart have been reported in several disease models, such as myocardial infarction and pressure overload or Ang II‐induced cardiac remodeling.^3^, ^4^, ^5^ Under physiological conditions, platelets circulate in a quiescent state. Platelets are sensitive in response to endothelial cell damage, sheer stress, abnormal hemodynamic conditions, etc.^6^, ^7^ Hypertensive patients are at a higher risk for thrombotic events, indicating platelets are activated.^8^, ^9^, ^10^ However, the precise mechanisms responsible for platelet activation and its role in mediating vascular inflammation, which are independent actions of hemostasis and vascular thrombosis, are very limited.

In the present study, we used a common anti‐platelet drug, clopidogrel, to study the role of platelets in promoting vascular inflammation and remodeling under the condition of Ang II infusion. Our results provide a novel mechanism for platelet‐mediated vascular inflammation and remodeling in response to Ang II stimulation.

## Methods

The data, analytic methods, and study materials will not be made available to other researchers for purposes of reproducing the results or replicating the procedure. The institutional review board of Dalian Medical University approved all of the studies.

### Animal Model and Drug Interventions

All animal care and experimental procedures were approved by the Animal Care and Use Committee of Dalian Medical University. Wild‐type male mice (C57BL/6, 10‐weeks‐old) were infused with Ang II at 490 ng/kg per minute with osmotic pumps (Alzet Model 1007D) for 1, 3, 7, or 14 days as described.^11^ Blood pressure was monitored by the tail‐cuff method. The control group was only infused with saline.

For antiplatelet treatment assay, mice were randomly assigned into 2 groups and 1 group received clopidogrel treatment 1 hour after infusion. Tablets containing clopidogrel (75 mg, Plavix; Sanofi‐Aventis) were ground into fine powder and freshly made in an emulsion in 0.5% methyl cellulose solution and administered by gavage. A loading dose of 15 mg/kg was given at the first day of treatment and then followed by a maintenance dose of 5 mg/kg once daily for the rest of the days.^3^ The nontreated group was only given vehicle (0.5% methyl cellulose solution).

To explore the mechanism of platelet activation following Ang II infusion, we treated mice with the angiotensin‐converting enzyme inhibitor (ACEI) perindopril (Servier, 6 mg/kg per day),^12^ angiotensin receptor blocker (ARB) olmesartan (Daiichi Sankyo Company Limited, 10 mg/kg per day)^13^ or vehicle by gavage, respectively.

### Bleeding Time Measurement

Inhibition of platelet activity by drug interventions was tested by measuring tail bleeding time as previously reported.^3^ Both treated and nontreated mice were subjected to bleeding time measurements. Sterile saline was poured into a 50‐mL test tube and heated in a 37°C water bath for 1 hour. A 10‐mm segment of the tail tip was cut off and the tip of the tail was immersed in prewarmed saline (37°C). Bleeding time was defined as the period of time where there was a clearly visible stream of blood that was continuously flowing. No visible blood flow was recognized as the termination of bleeding. The test was stopped at 20 minutes to prevent death caused by excessive blood loss in clopidogrel‐treated mice.

### Vascular Relaxation Studies

After 14 days’ infusion, rings from the descending aorta were obtained and cut into 4‐mm segments as previously described.^14^ Rings were transferred to an organ bath containing Krebs‐Henseleit solution (in mM: 110.8 NaCl, 5.9 KCl, 25.0 NaHCO_3_, 1.07 MgSO_4_, 2.49 CaCl_2_, 2.33 NaH_2_PO_4_, and 11.51 glucose, pH 7.4) maintained at 37°C, and then resting tension was adjusted stepwise to reach 0.5 g and allowed to stabilize for 1 hour. After stimulation by noradrenaline, vascular responses to increasing concentrations of acetylcholine and sodium nitroprusside were recorded. Mechanical activity was recorded by using a force transducer (Power Laboratory, AD Instruments) connected to a recorder (Power Laboratory, AD Instruments).

### Determination of Platelet Activation in Mouse

Mouse blood samples were collected by cardiac puncture at days 1, 3, and 7 after Ang II or saline infusion. Caution was taken to minimize agitation during withdrawal and the initial portion of blood (≈0.1 mL) was discarded. To obtain platelet‐rich plasma, blood (1 mL) was mixed with 300 μL of plateletwashing buffer (pH 6.5, 4.3 mm K_2_HPO_4_, 4.3 mm Na_2_HPO_4_, 24.3 mm NaH_2_PO_4_, 113 mm NaCl, 5.5 mm glucose, 0.5% bovine serum albumin, 10 mm theophylline) and then centrifuged at 250*g* for 20 minutes.^15^ Platelet pellets from platelet‐rich plasma were collected by centrifugation at 2000*g* for 2 minutes and resuspended in 2% fetal bovine serum/PBS. Phycoery‐conjugated P‐selectin antibody and fluorescein isothiocyanate–conjugated CD41 antibody (BD Biosciences) were added to 10 μL of platelet‐rich plasma and then incubated for 30 minutes in darkness. Isotype antibodies were used in the same concentrations as the detection antibodies.^3^ Samples were analyzed with a Becton‐Dickinson FACSCalibur flow cytometer.

### Detection of Platelet‐Leukocyte Binding

Platelet‐monocyte (P‐M) binding in blood was examined by flow cytometry. Blood samples were collected into a tube containing heparin (500 U/mL) by cardiac puncture at days 1, 3, and 7 after Ang II or saline infusion. Caution was taken to minimize agitation during withdrawal and the initial portion of blood was discarded. Red blood cells (100 μL blood) were lysed using the lysing buffer (BD Biosciences) and removed after centrifugation (500*g* for 5 minutes). After washing, pellets were re‐suspended in 2% fetal bovine serum /PBS and then were labeled with PerCP‐conjugated anti‐mouse CD45 (BD Biosciences), APC‐conjugated anti‐mouse CD115 (BD Biosciences), PE‐conjugated anti‐mouse Ly‐6C, (BD Biosciences), and fluorescein isothiocyanate–conjugated anti‐mouse P‐selectin (BD Biosciences) for 30 minutes in darkness. Isotype antibodies were used in the same concentrations as the detection antibodies.^3^ Platelets were identified as P‐selectin^+^. Leukocytes were identified as CD45^+^. Monocytes were identified as CD115^+^Ly‐6C^+^; neutrophils were identified as CD115^‐^Ly‐6C^+^; lymphocytes were identified as CD115^−^Ly‐6C^−^.^16^

Platelet‐macrophage binding was also examined in vessels. Briefly, tissues were minced into multiple small pieces and digested in an enzyme mixture, including collagenase type I (0.125 mg/mL) and type XI (0.05 mg/mL), hyaluronidase (0.025 mg/mL), DNase I (0.01 mg/mL) for 30 minutes at 37°C. The cell suspension was resuspended in 100 μL PBS and then incubated with PerCP‐conjugated anti‐mouse F/480 antibody (BD Biosciences) and fluorescein isothiocyanate–conjugated anti‐mouse P‐selectin antibody (BD Biosciences) for 30 minutes in the dark. After staining, samples were analyzed with the FACSCalibur flow cytometer.

### Histological Analysis

Vessels were fixed in phosphate‐buffered 4% formalin for 24 hours and then embedded in paraffin. Sections (5 μm) were examined by H&E and Masson's trichrome as described.^4^, ^5^ Images were viewed and captured using a microscope (Olympus).

### Immunohistochemistry

Immunohistochemistry was used to determine the contents of platelets (CD41) or macrophage infiltration (Mac‐3) in the vessel sections. Briefly, antigen retrieval was conducted by immersing in the citrate‐EDTA buffer and then in a microwave oven for 5 minutes at high power. Nonspecific staining was blocked by using 10% goat serum. After blocking, 50 μL of diluted primary antibodies (CD41, BD Biosciences; Mac‐3, Proteintech) was applied onto each section for 1 hour. Mouse IgG isotype control antibody (Jackson ImmunoResearch) was used at the same concentration as primary antibodies. After incubation with secondary antibody, sections were incubated with DAB until the desired staining was developed. Sections were then counterstained with Myer's hematoxylin for 2 minutes, then dehydrated and mounted with DePex.

### Dihydroethidine Staining

Frozen vessel sections were stained with the dihydroethidine (1 μmol/L in PBS) for 30 minutes at 37°C. Green autofluorescence and red dihydroethidine fluorescence were detected using a microscope (Olympus).

### Western Blotting

Cells or tissues were collected and total protein was isolated. Western blotting was performed with primary antibodies for CD41 (BD Biosciences), NADPH oxidase 1 (NOX1), NOX2, and NOX4 (Abcam). Membranes were re‐probed with GAPDH or tubulin to verify loading consistency.

### Real‐Time Polymerase Chain Reaction

Vessels or cells were collected for gene expression of inflammatory mediators by real‐time polymerase chain reaction. The primers used are listed in the Table. The cycling conditions consisted of an initial, single cycle of 5 minutes at 95°C, followed by 30 cycles of 30 s at 95°C, 30 s at 54°C, and 15 s at 72°C. The gene expression levels were quantified relative to the expression of 18s.

**Table 1:** Sequences of Primers

Gene	Primer
18s	
Forward	5′‐TTG ACG GGA AGG GCA CCA CCA G‐3′
Reverse	5′‐GCA CCA CCA CCC ACG GAA TCG‐3′
TNF‐α	
Forward	5′‐CTG TAG CCC ACG TCG TAG C‐3′
Reverse	5′‐TTG AGA TCC ATG CCG TTG‐3′
IL‐1β	
Forward	5′‐TTG ACG GAC CCC AAA AGA T‐3′
Reverse	5′‐GAA GCT GGA GCT CTC CAT CTG‐3′
IL‐6	
Forward	5′‐GCT ACC AAA CTG GAT ATA ATC AGG A‐3′
Reverse	5′‐CCA GGT AGC TAT GGT ACT CCA GAA‐3′
VCAM‐1	
Forward	5′‐TGG TGA AAT GGA ATC TGA ACC‐3′
Reverse	5′‐CCC AGA TGG TGG TTT CCT T‐3′

IL‐1β indicates interleukin‐1β; IL‐6, interleukin‐6; TNF‐α, tumor necrosis factor‐α; VCAM‐1, vascular cell adhesion molecule 1.

### Enzyme‐Linked Immunosorbent Assay

Plasma levels of cytokines and chemokines including interleukin‐1β (IL‐1β), IL‐6, IL‐12, monocyte chemotactic protein 1, and tumor necrosis factor‐α (TNF‐α) from mice were measured by ELISA according to the manufacturer's instructions.

### Platelets and Monocytes Co‐Culture

THP‐1 cells were grown in RPMI1640 containing 10% fetal bovine serum and 1% penicillin/streptomycin at 37°C in 5% CO_2_ incubator. Platelets were extracted and then co‐cultured with THP‐1 cells at 100:1 ratio for 24 hours following vehicle or Ang II treatment. After 24 hours, monocytes were collect and then assessed by real‐time polymerase chain reaction for inflammatory cytokines.

### Monocyte Adhesion Assay

Human umbilical vein endothelial cells were extracted from umbilical veins. Briefly, 20‐cm umbilical cords were stored in PBS containing gentamycin. Subsequently, cells were detached with 0.1% type II collagenase solution. Then cell suspension was collected in a 50‐mL tube and centrifuged for 15 minutes at 400 *g*. The cell pellet was resuspended in extracellular matrix (ECM) medium containing 10% fetal bovine serum and 1% penicillin/streptomycin. Human umbilical vein endothelial cells in serum‐free DMEM (100 μL) were stained by MitoGreen (green fluorescence) and plated to the upper chamber of a transwell chamber (8.0‐μm‐diameter pore, Corning) at 37°C in 5% CO_2_ for 24 hours. After 24 hours, THP‐1 cells were pre‐stained by DiI (red fluorescence) and co‐cultured with or without normal platelets in the human umbilical vein endothelial cells precoated upper chamber for 4 hours after vehicle or Ang II stimulation. After washing, the adhesive THP‐1 cells were detected by a fluorescence microscope (Olympus BX50).

### Data and Statistical Analysis

Data were expressed as means± SD. Data were analyzed using GraphPad Prism 7.0 software (GraphPad Software), using 1‐way ANOVA with Tukey's post hoc test and 2‐way ANOVA. Differences were considered statistically significant at *P* < 0.05.

## Results

### Ang II Infusion‐Induced Platelet Activation and Accumulation Within the Vessel

**Figure 1:**
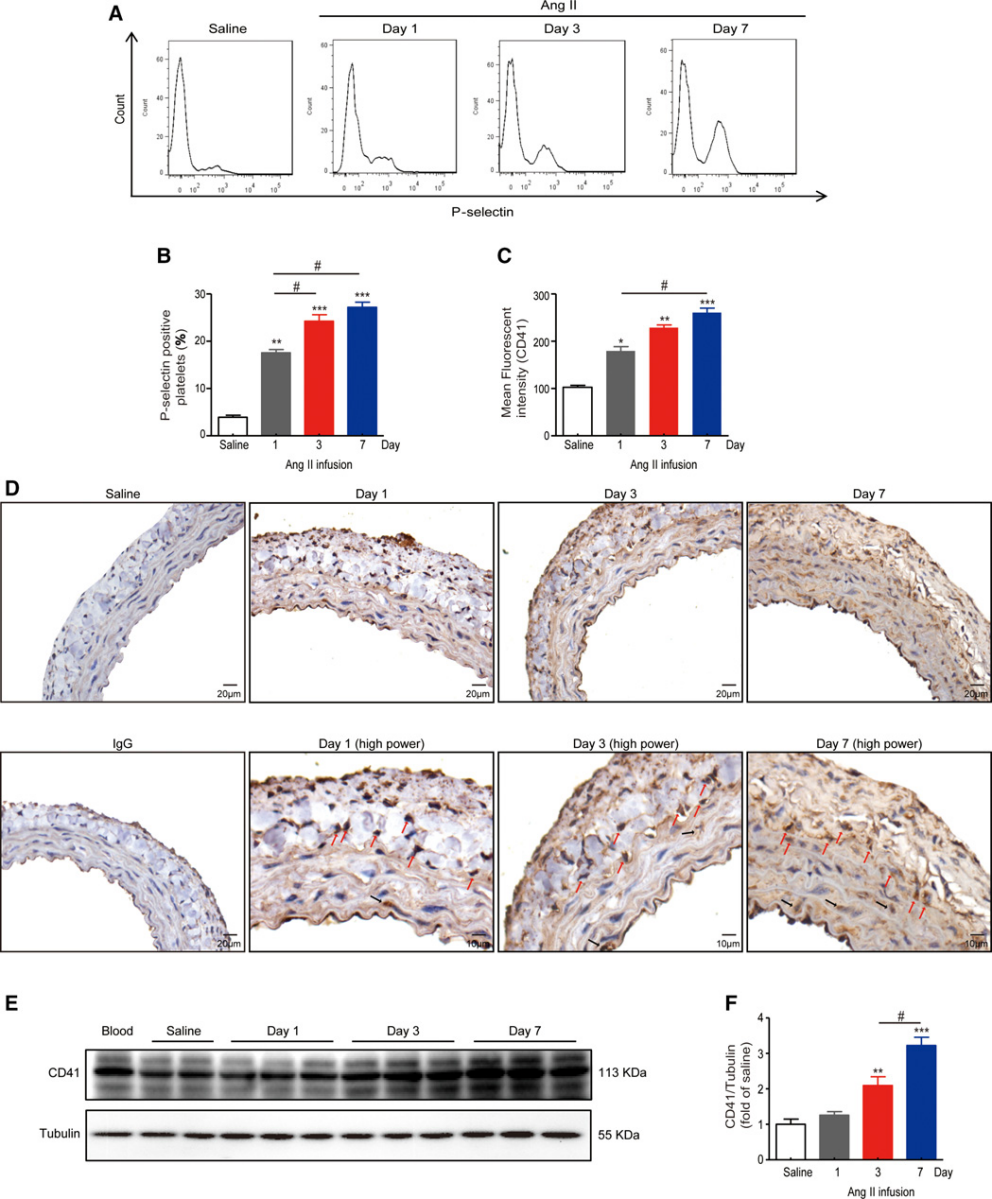
Ang II infusion induced platelet activation and accumulation within the vessel. A, Representative images of flow cytometry displaying activated platelets, determined by the population of platelets expressing P‐selectin relative to the whole population of platelets in the fresh blood from saline‐infused or Ang II‐infused vessels at days 1, 3, or 7, respectively. B, Quantitation of P‐selectin‐positive platelets. C, Quantitation of mean fluorescent intensity of CD41. D, Representative images of IHC staining showing platelet accumulation (CD41) within the vessels with saline infusion or Ang II infusion at days 1, 3, and 7. Black arrow indicates aggregates of platelets, while red arrow indicates colocalization of platelets and nuclei. E and F, Western blot analysis for the abundance of platelets (CD41) in purified platelets (first lane, positive control) or vessels from mice subjected to Ang II or saline. Ang II indicates angiotensin II; IHC, immunohistochemistry. *P<0.05, **P<0.01, ***P<0.001 vs saline; #*P* < 0.05; n=4 to 6 per group.

The proportion of circulating P‐selectin‐positive platelets increased significantly following Ang II infusion at days 1, 3, and 7 (3.9±0.9% versus 17.6±1.5%, 24.3±3.0%, 27.2±2.4%), indicating platelets were activated (Figure 1A and 1B). Meanwhile, the content of CD41, as indicated by mean fluorescent intensity of FL2 (102.8±4.0 versus 179.3±9.5, 228.8±5.8, 260.4±9.8), was also increased in response to Ang II stimulation (Figure 1C). Time‐dependent accumulation of platelets within the vessel was observed at days 1, 3, and 7 after Ang II infusion, as assessed by the platelet‐specific marker CD41 (Figure 1D). Platelets were largely colocalized with dense nuclei in either media or adventitia. Western blot further confirmed platelets were deposited in the vessel following Ang II infusion (1.0±0.1 versus 1.3±0.1, 2.1±0.2, 3.2±0.2) (Figure 1E and 1F).

### Clopidogrel Abolished Ang II‐Induced Platelet Activation, Vascular Inflammatory Responses, and Remodeling

**Figure 2:**
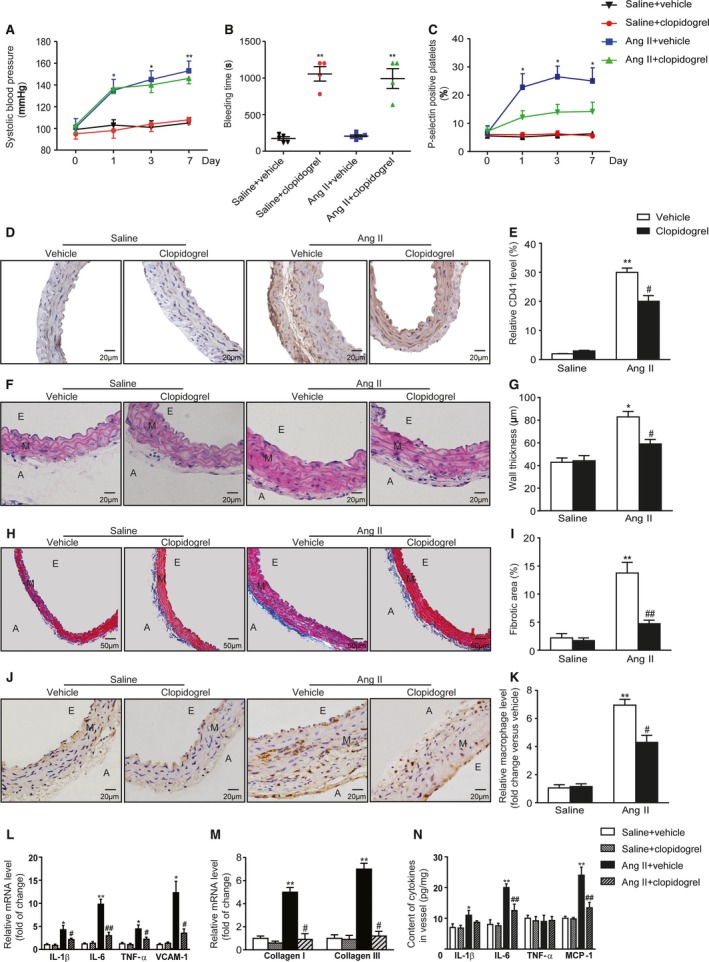
Clopidogrel abolished Ang II‐induced platelet activation, and vascular inflammatory responses and remodeling. A through C, systolic blood pressure, bleeding time, and P‐selectin of groups with saline or Ang II infusion, with or without clopidogrel treatment. D, Representative images of IHC staining of CD41 in the vessel sections. E, Quantitation of CD41. F, Representative images of H&E staining of the vessels. G, Wall thickness of each group was analyzed. H, Representative images of Masson staining of the vessels. I, The percentage of fibrotic area was analyzed. J, Representative images of IHC staining of Mac‐3 on vessel sections. K, Mac‐3 positive cells were analyzed. L, mRNA levels of IL‐1β, IL‐6, TNF‐α, and VCAM‐1 in vessels. M, mRNA levels of collagen I and III in vessels. N, Plasma levels of IL‐1β, IL‐6, TNF‐α, and MCP‐1. A indicates adventitia; Ang II, angiotensin II; E, endangium; IHC, immunohistochemistry; IL‐1β, interleukin‐1β; IL‐6, interleukin‐6; M, media; MCP‐1, monocyte chemotactic protein 1; TNF‐α, tumor necrosis factor‐α; VCAM‐1, vascular cell adhesion molecule 1. *P<0.05, **P<0.01 vs saline+vehicle; ^#^ *P*<0.05, ^##^ *P*<0.01 vs Ang II+vehicle; n=4 to 6 per group.

Ang II led to an increase in systolic blood pressure from a baseline of 100 mm Hg to 152 mm Hg after 7 days’ infusion, and such increasing was not abolished by clopidogrel therapy (152±9 mm Hg versus 146±5 mm Hg) (Figure 2A). As expected, clopidogrel treatment significantly prolonged tail bleeding time in mice with both saline and Ang II infusion (Figure 2B). Ang II infusion stimulated circulating platelet activation and deposition in the vessel, which was inhibited by clopidogrel treatment (25.0±4.7% versus 14.2±3.3%; 28.7±2.4% versus 18.3±2.9%) (Figure 2C through 2E). Furthermore, Ang II‐induced vascular wall thickening and collagen deposition was significantly attenuated by clopidogrel treatment (82.8±4.8 μm versus 59.0±4.0 μm; 13.8±1.9% versus 4.7±0.6%) (Figure 2F through 2I). Moreover, Ang II‐induced severe inflammatory responses, which were characterized by vascular macrophage infiltration and upregulation of pro‐inflammatory mediators in both vessel and blood, were also markedly suppressed by antiplatelet intervention (Figure 2J through 2N). Thus, we believe that platelet activation and vascular accumulation is an early event upon Ang II stimulation, and associated with vascular and systemic inflammatory responses.

### Clopidogrel Treatment Reduced Vascular Oxidative Stress in Response to Ang II

**Figure 3:**
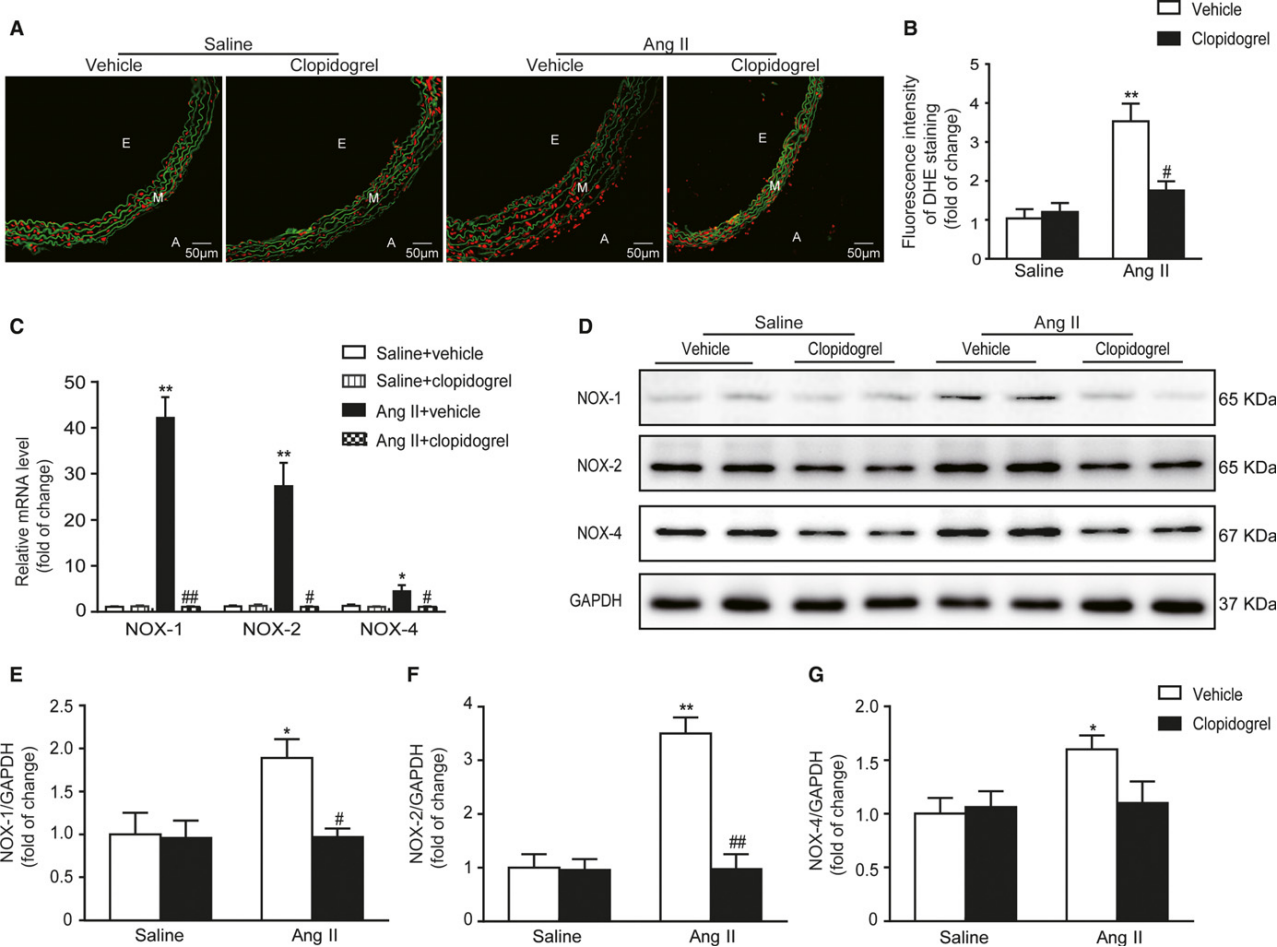
Clopidogrel reduced vascular oxidative stress in response to Ang II. A, Dihydroethidine staining of vessels with or without clopidogrel treatment following saline or Ang II infusion. Oxidative stress was stained by dihydroethidine, which was indicated by red fluorescence. The elastic lamina was indicated by green fluorescence. B, The fluorescent intensity of dihydroethidine was analyzed. C, mRNA levels of NOX‐1, NOX‐2, and NOX‐4 in vessels. D, The protein levels of NOX‐1, 2, and 4 in vessels were examined by using Western blot. E through G, Quantitation of NOX‐1, 2, and 4. A indicates adventitia; Ang II, angiotensin II; DHE, dihydroethidine; E, endangium; M, media; NOX‐1, NADPH oxidase 1; NOX‐2, NADPH oxidase 2; NOX‐4, NADPH oxidase 4. E: endangium; M: media; A: adventitia. *P<0.05, **P<0.01 vs saline+vehicle; ^#^ *P*<0.05, ^##^ *P*<0.01 vs Ang II+vehicle; n=4 to 6 per group.

Ang II significantly increased vascular oxidative stress, as indicated by dihydroethidine staining, and this effect was abolished by clopidogrel therapy (3.5±0.5 versus 1.8±0.2) (Figure 3A and 3B). Furthermore, levels of vascular NOX1, NOX2, and NOX4 were significantly increased following Ang II infusion at both mRNA and protein levels, but this increase was markedly reduced after clopidogrel treatment (Figure 3C through 3G).

### Clopidogrel Treatment Suppressed Ang II‐Evoked P‐M Binding in Circulation and Vessel

**Figure 4:**
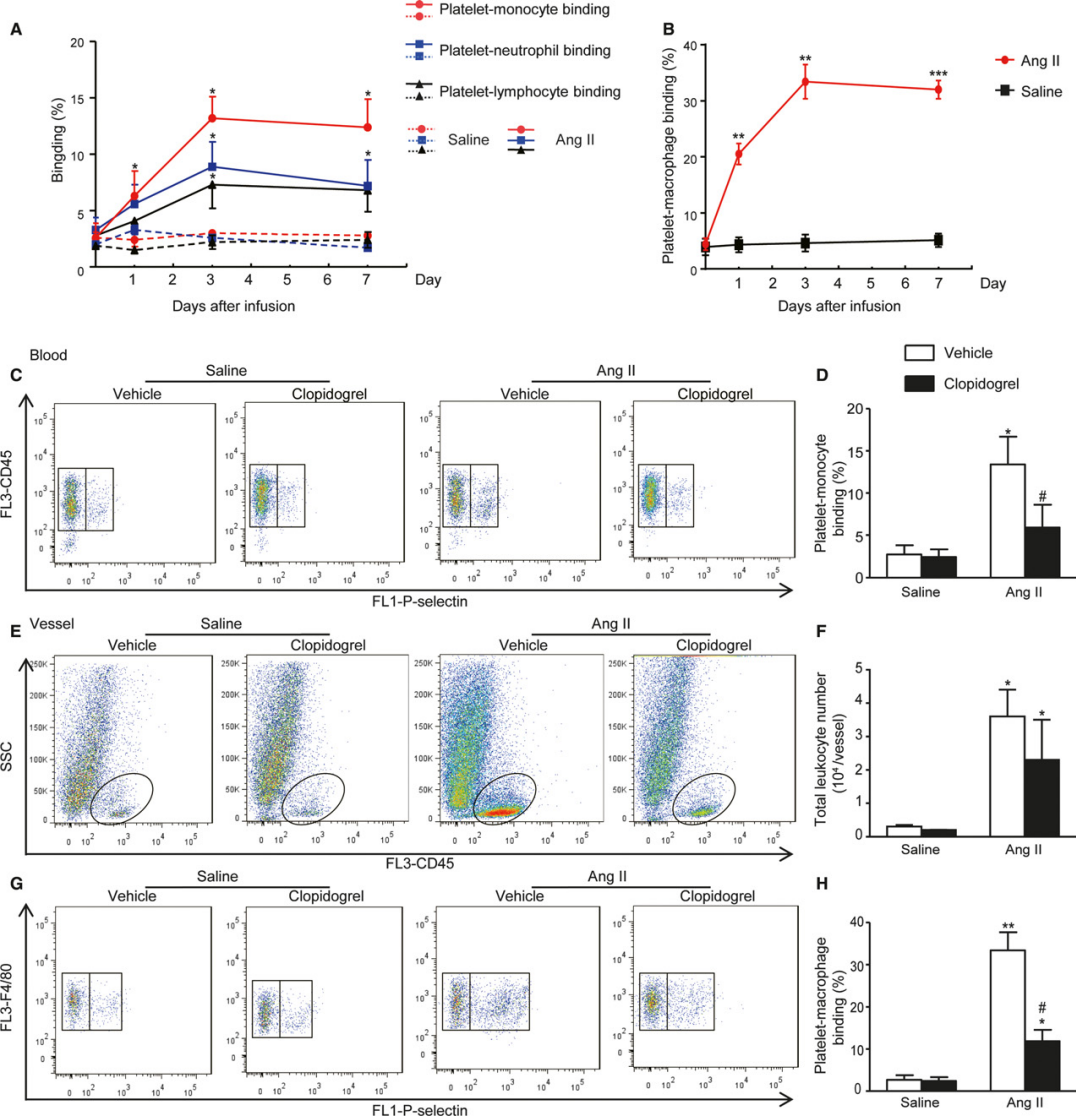
Clopidogrel suppressed Ang II‐evoked P‐M binding both in circulation and vessel. A, Time course of platelet‐monocyte, platelet‐neutrophil, and platelet‐lymphocyte binding in the blood of mice with saline or Ang II infusion determined by flow cytometry. B, Time course of platelet‐macrophage binding in vessels of mice with saline or Ang II infusion determined by flow cytometry. C, Representative images of flow cytometry displaying P‐M binding in blood, determined by the population of monocytes expressing P‐selectin relative to the whole population of monocytes. D, Quantitation of circulating P‐M binding. E, Representative images of flow cytometry displaying infiltrated leukocytes, determined by CD45‐positive cells in the vessels. F, Quantitation of infiltrated leukocytes in vessels. G, Representative images of flow cytometry displaying infiltrated P‐M binding in vessels, determined by the population of cells expressing P‐selectin and F4/80 relative to the whole population of F4/80 macrophages. H, Quantitation of infiltrated P‐M binding in the vessel samples. Ang II indicates angiotensin II; P‐M, platelet‐monocyte. *P<0.05, **P<0.01 vs saline+vehicle; ***P<0.001; ^#^ *P*<0.05 vs Ang II+vehicle; n=5 to 6 per group.

Platelets bind with inflammatory cells that are critical for initiation of inflammation. We therefore examined the effect of platelets binding to different inflammatory cells. As shown in Figure 4A, Ang II induced a markedly increasing in P‐M binding as early as day 1, which progressively increased during the whole period of Ang II infusion (2.6±1.3% versus 6.3±2.2%, 13.2±3.8%, 12.8±3.7%). Platelets binding with neutrophils (3.3±1.1% versus 5.6±1.7%, 8.9±2.2%, 7.2±2.3%) and lymphocytes (2.8±0.9% versus 4.1±1.6%, 7.3±2.1%, 6.8±1.9%) was also significantly increased at days 1, 3, and 7 (Figure 4A). Meanwhile, the increasing in P‐M binding was also observed in the vessel, with a trend similar to the circulating level (4.4±1.3% versus 20.5±4.2%, 33.4±6.8%, 28.4±3.7%) (Figure 4B). Clopidogrel therapy largely blunted Ang II‐induced P‐M binding both in blood (13.4±3.3% versus 5.9±2.7%) and vessels (33.4±4.3% versus 11.9±2.7%), without influencing the baseline level at day 7 (Figure 4C, 4D, 4G and 4H). Notably, clopidogrel therapy also inhibited inflammatory cell infiltration into the vessel (Figure 4E and 4F).

### Platelets Promote Monocyte Activation in Response to Ang II Stimulation

**Figure 5:**
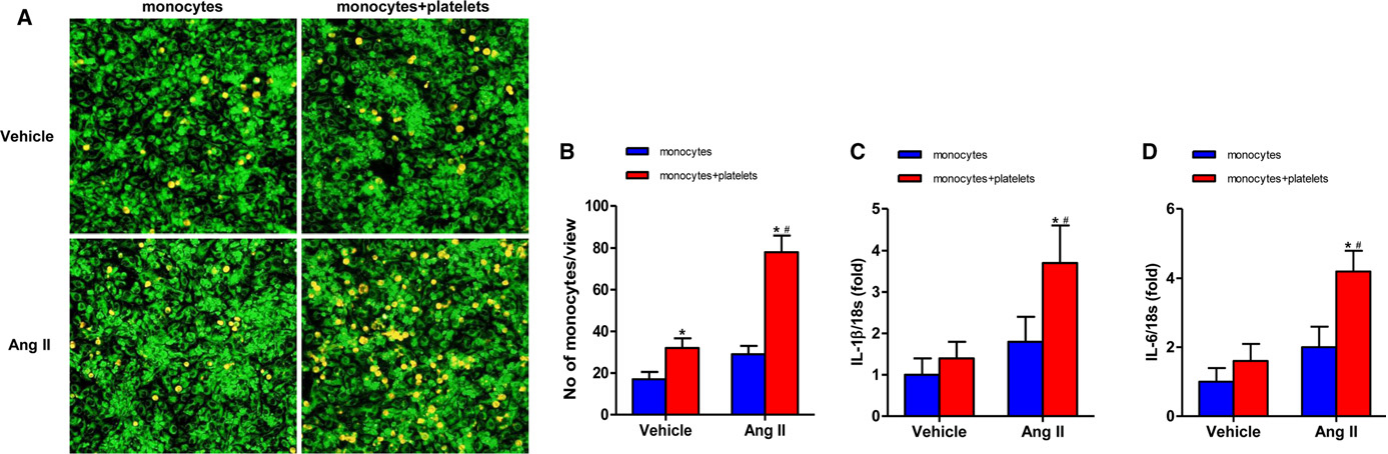
Platelets promoted monocyte activation in response to Ang II stimulation. A, Representative images showed adhesive monocytes to HUVECs under the different conditions. HUVECs were stained by MitoGreen, determined by green fluorescence. THP‐1 cells were stained by Dil, determined by red fluorescence. B, Quantitation of adhesive monocytes. C and D, mRNA levels of IL‐1β and IL‐6 in vessels. Ang II indicates angiotensin II; IL‐1β, interleukin‐1β; IL‐6, interleukin‐6; HUVECS, human umbilical vein endothelial cells. *P<0.05 vs vehicle+monocytes alone; ^#^ *P*<0.05 vs Ang II+monocytes alone; n=5 to 6 per group.

To investigate whether monocytes were activated following P‐M binding, we co‐cultured platelets and monocytes. In the co‐culture condition, Ang II enhanced the monocyte activation, as compared with monocytes cultured alone, which was measured by the adhesive ability (78±8 versus 29±4) (Figure 5A and 5B) and the content of IL‐1β (3.7±0.9 versus 1.8±0.6) and IL‐6 (4.2±0.6 versus 2.0±0.6) in mRNA levels (Figure 5C and 5D). Vehicle lost the ability to induce monocyte activation.

### Clopidogrel Treatment Prevented Vascular Dysfunction in Response to Ang II

**Figure 6:**
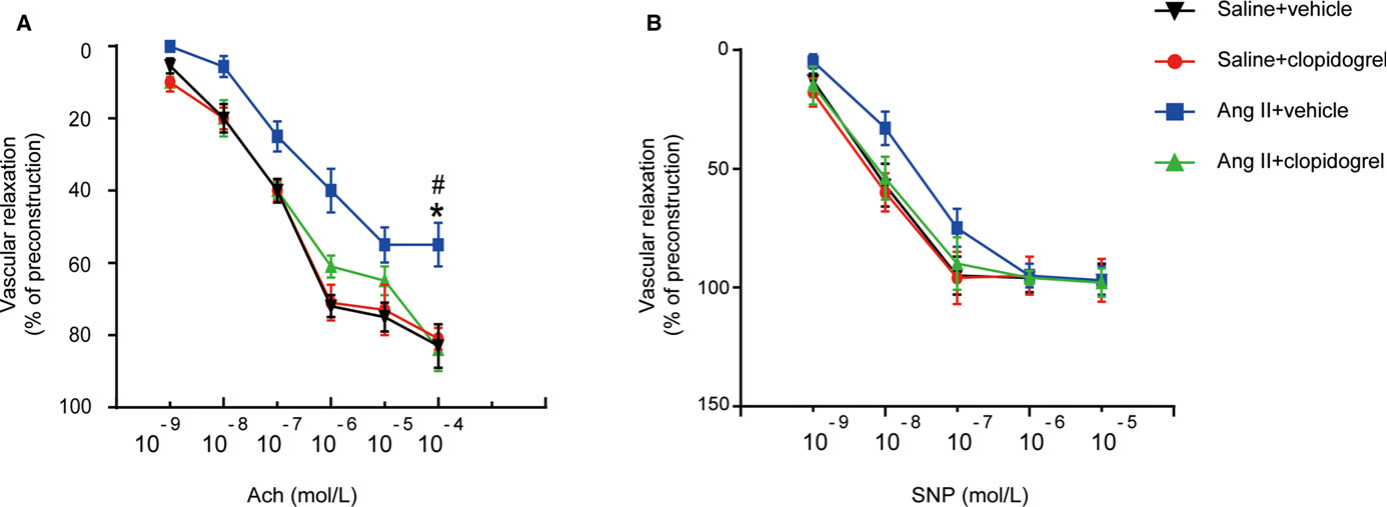
Clopidogrel treatment prevented vascular dysfunction in response to Ang II. A, Concentration‐response curves of endothelium‐dependent (Ach) in mice with saline or Ang II infusion, with or without clopidogrel treatment. B, Concentration‐response curves of endothelium‐independent (SNP) relaxation in mice with saline or Ang II infusion, with or without clopidogrel treatment. Ang II indicates angiotensin II; Ach, acetylcholine; SNP, sodium nitroprusside. *P<0.05 vs saline+vehicle; ^#^ *P*<0.05 vs Ang II+clopidogrel; n=5 to 6 per group.

To examine how platelet inhibition ameliorated vascular dysfunction, we measured ex vivo vascular function in vehicle or clopidogrel‐treated mice infused with either saline or Ang II at day 14. The whole vessels were isolated from mice, and concentration‐relaxation curves in response to acetylcholine or sodium nitroprusside were examined. Ang II infusion significantly impaired endothelium‐dependent vasodilatation to acetylcholine, whereas these effects were markedly preserved in clopidogrel‐treated mice (55.0±5.5% versus 84.0±6.0%) (Figure 6A). Ang II infusion only mildly affected endothelium‐independent vasodilatation to sodium nitroprusside in Ang II‐infused mice as compared with vehicle control, but there was no significant difference (97.0±5.0% versus 98.0±6.0%) (Figure 6B). Clopidogrel treatment had no effect on acetylcholine and sodium nitroprusside–induced vasodilatation under saline infusing condition (Figure 6A and 6B). These results indicate that inhibiting platelet activation prevented Ang II‐induced vascular dysfunction.

### Effects of ARB and ACEI on Platelet Activation, P‐M Conjugation, and Vascular Accumulation in Mice With Ang II Infusion

**Figure 7:**
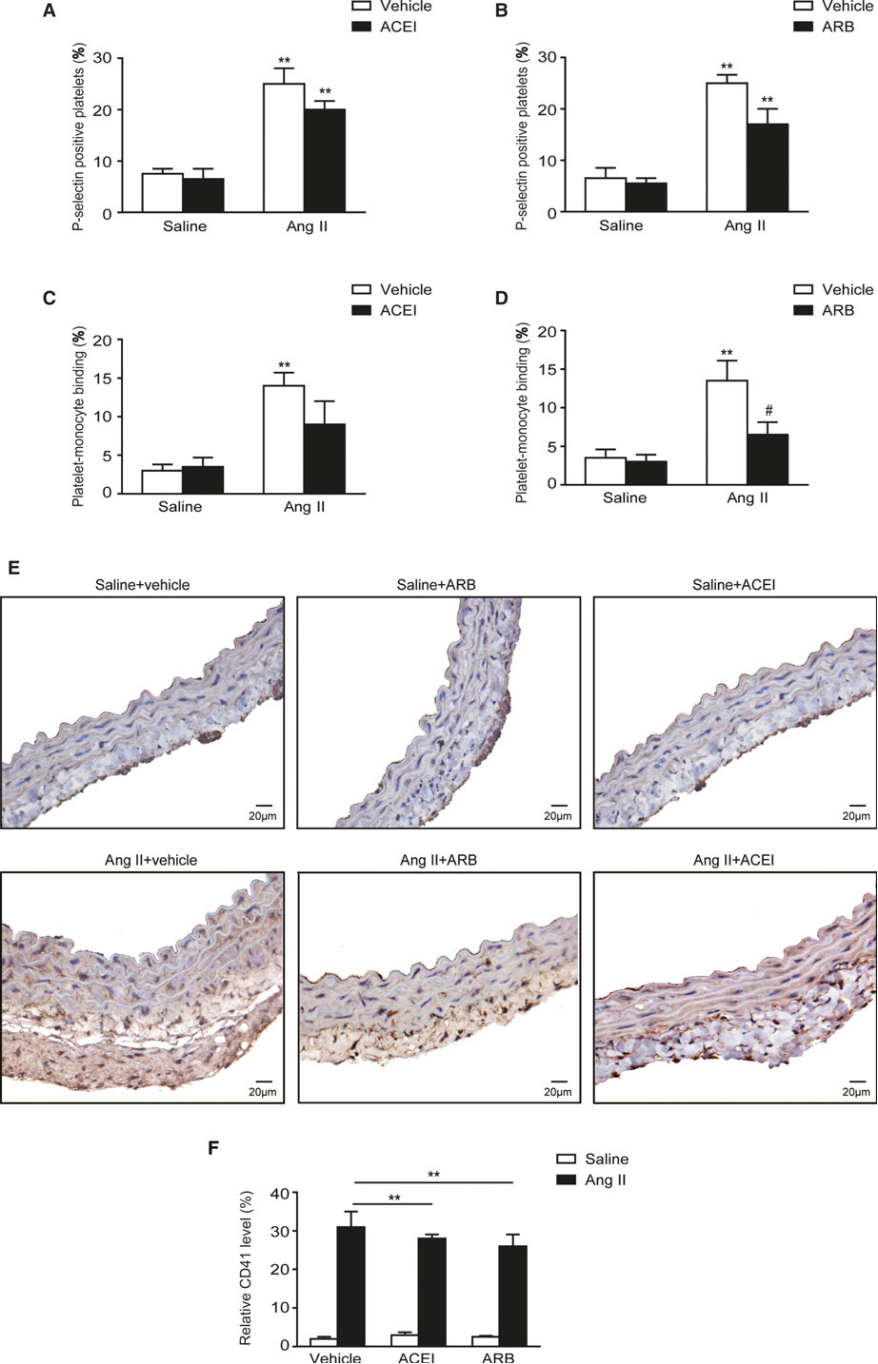
Effects of ACEI and ARB on platelet activation and vascular accumulation in mice with Ang II infusion. A, Quantitation of P‐selectin‐positive platelets in the blood from mice with saline or Ang II infusion, with or without ACEI treatment. B, Quantitation of P‐selectin‐positive platelets in the blood from mice with saline or Ang II infusion, with or without ARB treatment. C, Quantitation of circulating P‐M binding in the blood from mice with saline or Ang II infusion, with or without ACEI treatment. D, Quantitation of circulating P‐M binding in the blood from mice with saline or Ang II infusion, with or without ARB treatment. E, IHC staining of platelets in vessel sections of mice with or without ARB/ACEI treatment in response to saline or Ang II infusion. F, Quantitation of platelet accumulation in vessel samples. Ang II indicates angiotensin II; ACEI, angiotensin‐converting enzyme inhibitor; ARB, angiotensin receptor blocker; IHC, immunohistochemistry; P‐M, platelet‐monocyte. **P<0.01 vs saline+vehicle; ^#^ *P*<0.05 vs Ang II+vehicle; n=4 to 6 per group.

To test the effect of routinely used antihypertensive drugs on platelet parameters, we treated mice with olmesartan (ARB) or perindopril (ACEI), and then measured P‐selectin‐positive platelets and P‐M conjugation in circulating blood as well as platelet accumulation in the vessels at day 7 after Ang II infusion. Treatment with olmesartan and perindopril both mildly suppressed the platelet activation, but no significant differences were found (Figure 7A and 7B). Olmesartan significantly abolished P‐M binding in blood (13.5±2.6% versus 6.5±1.6%) (Figure 7C and 7D). Both olmesartan and perindopril were effective in suppressing vascular platelet accumulation (Figure 7E and 7F).

### Potential Role of Platelets in Ang II‐Infused Vessel

**Figure 8:**
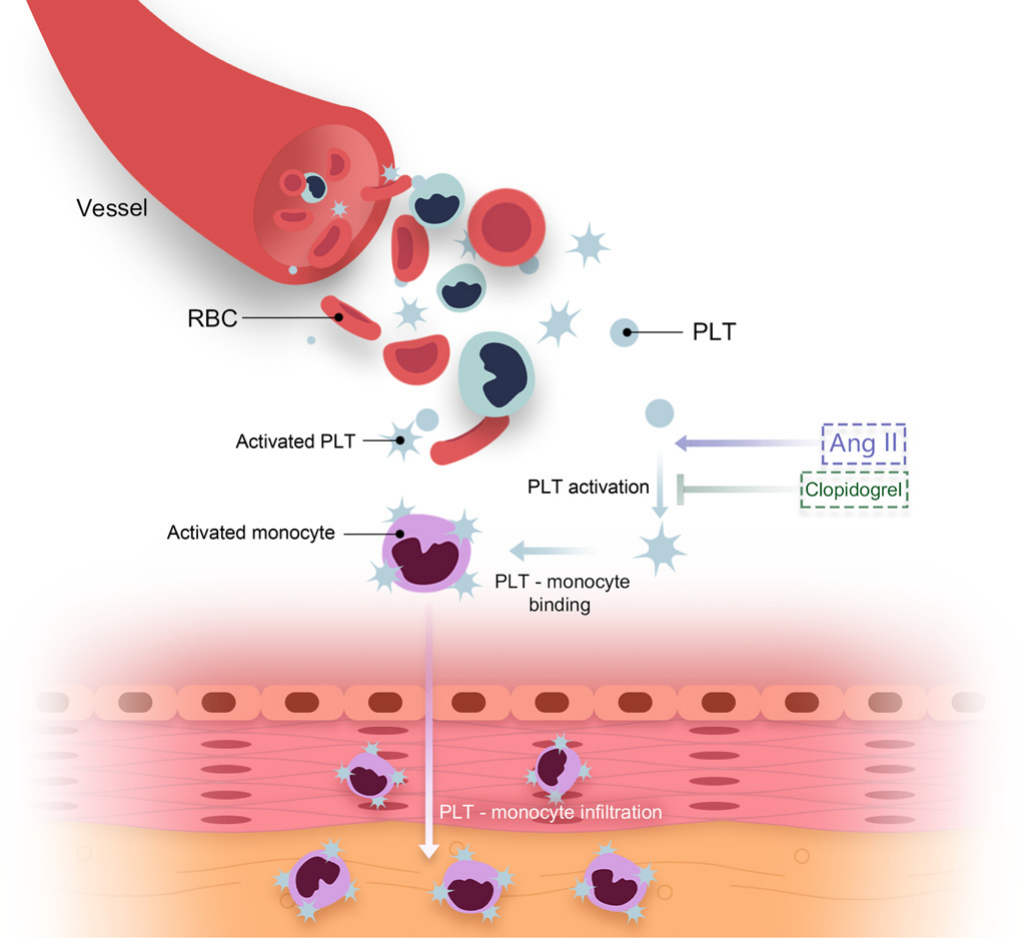
The potential role of platelets in Ang II‐infused vessel. High levels of Ang II induced platelet activation. Activated platelets subsequently bind to monocyte in the circulation, promoting monocyte activation. P‐M binding infiltrates into the vessel and then enhances vascular inflammatory responses. Ang II indicates angiotensin II; P‐M, platelet‐monocyte.

A high level of Ang II induces platelet activation. Activated platelets subsequently bind to monocytes in the circulation, promoting monocyte activation. P‐M binding infiltrates into the vessel and then enhances vascular inflammatory responses (Figure 8).

## Discussion

In the present study, several novel findings have been made: (1) Platelets were accumulated in the vessels following Ang II infusion; (2) Binding of circulating platelets with monocytes were elevated both in blood and vessels in the setting of Ang II infusion; (3) Clopidogrel markedly reduced platelet vascular deposition, suppressed inflammatory responses and oxidative stress, as well as attenuated vascular remodeling and dysfunction following Ang II stimulation; (4) Clopidogrel suppressed Ang II‐induced P‐M binding both at circulating and regional levels. Our findings indicate that platelets play a critical role in Ang II‐induced vascular inflammation and remodeling, with a marked attenuation of P‐M binding as an important mechanism, and reveal additional efficacy of antiplatelet interventions for Ang II‐induced mice (Figure 8).

Platelets have been shown to localize in heart tissues under myocardial infarction, Ang II, or pressure overload–induced conditions.^3^, ^4^, ^5^ Consistently, we found platelets accumulated in the vessels following Ang II stimulation. How do platelets migrate to the vessel? According to our immunohistochemistry, we found that some aggregates of platelets were visible in the media of the vessel, which were likely caused by vessel damage or increased vascular permeability. In addition, platelets were colocalized with dense of nuclei in both media and adventitia of the vessel, and such nuclei were likely infiltrated inflammatory cells. Thus, we hypothesized that platelets might enter into the vessel by binding with leukocytes. Furthermore, we confirmed that P‐M binding was present in both circulation and vessels in response to Ang II stimulation by using flow cytometry, which strengthened our hypothesis.

As indicated by our findings, we believe that platelets contribute to vascular inflammatory responses via 2 mechanisms. First, platelets are a rich cellular source of chemokines, cytokines, and growth factors. Activated platelets might release inflammatory mediators to the circulation and vessel,^17^, ^18^, ^19^ which promote endothelial cell damage 20, 21 or vascular smooth muscle cell proliferation.^22^, ^23^ Second, platelets also contribute to inflammation via platelet‐leukocyte binding, likely driven by P‐selectin on the activated platelets and P‐selectin glycoprotein ligand‐1 on leukocytes.^3^, ^24^, ^25^ After binding with P‐selectin, P‐selectin glycoprotein ligand‐1 functions as a signaling molecule to induce leukocyte activation.^25^, ^26^ Platelet‐leukocyte binding has been found in the blood from patients or mice with myocardial infarction or pressure overload, which is associated with the degree of inflammation and disease progression.^3^, ^5^, ^27^, ^28^ Importantly, P‐M binding has been found in hypertensive patients,^29^, ^30^ showing a strong positive correlation with blood pressure.^29^ Renal denervation reduced P‐M binding in hypertensive patients.^30^ An important finding from our study is that increased platelet‐leukocyte binding, especially P‐M binding, present both in the blood and vessel following Ang II infusion, suggests that regional accumulated platelets were at least in part deposit in vessels by binding with infiltrated monocytes. We believe that platelet activation, formation, and vascular infiltration of P‐M binding might be a rapid and dynamic process in response to Ang II stimulation.^3^, ^4^, ^5^ Circulating P‐M binding could serve as a useful cellular marker both for the degree of vascular inflammation and systemic inflammation in hypertensive patients.

Thienopyridines are a class of ADP receptor (P2Y_12_) inhibitors used for suppression of platelet activity, of which clopidogrel has been well studied. In addition to potent antithrombotic efficacy, treatment with clopidogrel has shown valuable anti‐inflammatory effects on hearts from the mouse model of myocardial infarction, hypertrophy, and heart failure.^3^, ^4^, ^5^ Clinically, clopidogrel reduced serum levels of various inflammatory cytokines in patients who underwent stenting.^31^, ^32^ However, the beneficial effects of clopidogrel responsible for vascular inflammation remained unknown. Here, we observed attenuation of Ang II‐induced P‐M binding both in blood and vessels after clopidogrel treatment, associated with a reduced degree of inflammation. Further clinical studies will be required.

Monocytes/macrophages were shown to be responsible for Ang II‐induced vascular inflammation and dysfunction.^33^ In this study, our flow cytometry showed that platelets were preferred to bind with monocytes/macrophages. Furthermore, Ang II‐induced hypertension and vascular dysfunction are associated with increased vascular reactive oxidative stress (ROS) production.^34^ Although the exact cellular sources of ROS have not been defined, macrophages are believed to be the main source of ROS in the vessel.^35^ Inhibition of platelet activation effectively reduced the vascular levels of NADPH oxidase subunits such as NOX‐1, 2, and 4, and was associated with reduced infiltration of macrophages in the aorta in response to Ang II. Our findings suggested ROS generation was at least in part from infiltrated macrophages. Whether vascular accumulated platelets are the source of ROS remains to be examined.

Platelet activation was found in patients with hypertension, which was associated with increased cardiovascular events and mortality. Platelet activation is reduced after commencement of antihypertension therapies in hypertensive patients.^36^, ^37^ Consistent with these findings, we found platelet activation is an early event in Ang II infusion‐induced vascular inflammation. Ang II is involved in many biological functions, in addition to blood pressure regulation. Platelets expressed Ang II receptor type 1 (AT1 receptors) on the surface,^38^ and blockage of AT1 receptors reduced P‐selectin expression on platelets, and inhibited platelet adhesion to vessels in mice.^39^, ^40^ Thus, Ang II is able to induce platelet activation by activating AT1 receptor directly, albeit the detailed mechanism remains to be investigated. Furthermore, the classic mode of platelet activation is endothelial cell dependent.^41^ Ang II is capable of inducing endothelial cell damage,^42^ which might further induce platelet activation. Moreover, phenylephrine injection in mice is able to induce a mild increase in circulating P‐M binding,^4^ suggesting that high blood pressure could be another way to promote platelet activation.

ARB and ACEI are commonly used for the treatment of hypertension. Both ARB and ACEI are known to exhibit antiplatelet activity.^12^, ^39^, ^40^ Our individual testing of perindopril and olmesartan revealed similar effects of antihypertension drugs in inhibiting P‐M binding and platelet regional accumulation. Thus, inhibition of platelet activation by these agents could be of additional benefit in hypertensive patients and are desirable to treat hypertensive patients with high inflammation status and/or thromboembolic risk.

In conclusion, we demonstrated that clopidogrel was effective in suppressing Ang II‐induced vascular inflammation and remodeling, by attenuating platelet activation and P‐M binding. P‐M binding could be the new therapeutic targets or as a biomarker for vascular inflammation severity and clinical outcomes in hypertensive patients.

## Sources of Funding

Liu was supported by the Talent Foundation of the First Affiliated Hospital of Dalian Medical University (2017D044). Wang was supported by a grant from the Nature Science Foundation of Liaoning Province (20170540291). An was supported by Initial Funding for Doctorate Programme of Liaoning Province (20180540001).

## Disclosures

None.
